# The role of anxiety and related states in pediatric postsurgical pain

**DOI:** 10.1080/24740527.2020.1847600

**Published:** 2020-12-30

**Authors:** Cheryl H. T. Chow, Louis A. Schmidt, D. Norman Buckley

**Affiliations:** a Department of Psychology, Neuroscience & Behavior, McMaster University, Hamilton, Ontario, Canada; b Department of Anesthesia, McMaster University, Hamilton, Ontario, Canada

**Keywords:** pediatric acute postsurgical pain, pediatric chronic postsurgical pain, child anxiety, parent anxiety, catastrophizing, anxiety sensitivity, pain trajectories, pain anxiety, surgery

## Abstract

**Background:** Nearly 20% of children and adolescents have pain with disability 1 year after surgery, and they experience poor sleep, school absence, and decreased activities. Negative clinical, psychological, and developmental effects include greater pain medication use, longer recovery, and fear of future medical care. Research has found psychological and family influences (i.e., child and parental anxiety) on pediatric chronic postsurgical pain (CPSP), but a better understanding of the role of perioperative anxiety and its related states in predicting pediatric postsurgical pain is needed. The poor understanding of the causes of child CPSP can lead to misdiagnosis and inadequate treatment, with significant short- and long-term effects.

**Objectives:** The aim of this review was to summarize the literature on children’s perioperative anxiety and parental anxiety in relation to acute postsurgical pain, CPSP, and pain trajectories. We also examined other related psychological factors (i.e., anxiety sensitivity, catastrophizing, pain anxiety, and fear of pain) in relation to pediatric acute and chronic postsurgical pain. Lastly, we discuss the interventions that may be effective in reducing children’s and parents’ preoperative anxiety.

**Conclusions:** Our findings may improve the understanding of the causes of CPSP and highlight the gaps in research and need for further study.

## RÉSUMÉ

**Contexte:** Près de 20 % des enfants et adolescents ont des douleurs avec incapacité un après la chirurgie, et ils ont un mauvais sommeil, des absences scolaires et une diminution de leurs activités. Les effets cliniques, psychologiques et développementaux négatifs comprennent une utilisation accrue des analgésiques, une récupération plus longue et la peur des soins médicaux futurs. La recherche a révélé des influences psychologiques et familiales (c.-à-d. de l’anxiété chez les enfants et les parents) sur la douleur chronique postchirurgicale pédiatrique, mais une meilleure compréhension du rôle de l’anxiété périopératoire et ses états associés pour prédire la douleur postchirurgicale pédiatrique est nécessaire. Une mauvaise compréhension des causes de la douleur chronique postchirurgicale pédiatrique peut entraîner un diagnostic erroné et un traitement inadéquat, avec des effets importants à court et à long terme.

**Objectifs:** Le but de cette revue était de résumer la littérature sur l’anxiété périopératoire des enfants et l’anxiété parentale en lien avec la douleur postchirurgicale aiguë, la douleur chronique postchirurgicale et les trajectoires de la douleur. Nous avons aussi examiné d’autres facteurs psychologiques connexes (ex. : sensibilité à l’anxiété, catastrophisme, anxiété de la douleur, et peur de la douleur) en lien avec la douleur chronique postchirurgicale pédiatrique aigue. Enfin, nous discutons des interventions qui peuvent être efficaces pour réduire l’anxiété préopératoire des enfants et des parents.

**Conclusions:** Nos résultats peuvent améliorer la compréhension des causes de la douleur chronique postchirurgicale et mettre en évidence les lacunes dans la recherche, ainsi que la nécessité d’études plus poussées.

## Introduction

Approximately 5 million children and adolescents undergo surgery in the United States each year; among those undergoing major surgery, up to 20% experience moderate–severe postsurgical pain 3 months after surgery.^1^ The range of prevalence for pediatric chronic postsurgical pain (CPSP) has been found to be between 11% and 38%,^2,3^ with various pain trajectories illustrated by graphical representations of a patient’s pain scores over a period of observation.^3–5^

CPSP is defined as pain persisting for at least 3 months after surgery that is present in or referred from the surgical site and causes significant distress or impairment to quality of life. Further, to be considered CPSP, the pain should not be a continuation of previous pain, and other causes of the pain must be ruled out.^1^^,6–8^ Studies have also shown that up to 30% of youth with CPSP reported reduced functional ability up to 1 year after surgery, with interference in daily functioning, resulting in poorer short- and long-term outcomes such as decreased academic performance, poor peer relationships, and disruptions in sleep or daily activities.^9–11^ Overall, quality of life is greatly reduced among children and adolescents with CPSP.^10^

There is growing evidence implicating psychological factors such as pediatric perioperative anxiety, parental pain anxiety, and catastrophizing in the development of CPSP in the pediatric population, but a better understanding of the role of perioperative anxiety and its related states in predicting pediatric postsurgical pain is needed.^1,4,6,11,12^ Pain catastrophizing is a concept closely related to anxiety in the surgical context. It is defined as the tendency to magnify the threat value of pain, dwell on the negative associations to pain (rumination), and feel helpless in the context of pain; it is associated with both state and trait anxiety and with more specific pain-related fears.^13^ Thus, further understanding the role of anxiety and related states in pediatric acute (i.e., defined as pain occurring immediately after surgery and persisting for up to 30 days depending on the condition,^14^) and chronic postsurgical pain might potentially identify a modifiable factor affecting the course of postsurgical pain and to help to identify and treat children and adolescents who are at risk for CPSP. Therefore, it is imperative to conduct the present narrative review to provide a current state of knowledge on this issue to inform future research and practice in mitigating pediatric postsurgical pain.

## Aim

To gain a better understanding of the role of perioperative anxiety in predicting pediatric postsurgical pain, the aim of this narrative review was to summarize the literature on pediatric perioperative anxiety (state/trait) and parental anxiety (state/trait) in relation to pediatric acute postsurgical pain, CPSP, and CPSP trajectories. We also discuss other related psychological factors (i.e., anxiety sensitivity, pain catastrophizing, pain anxiety, and fear of pain) in relation to pediatric postsurgical pain.

## Background

The impact of postsurgical pain extends well beyond the immediate perioperative period. Children and adolescents with postsurgical pain report frequent use of pain medications, which might place them at risk of developing substance abuse problems in adulthood,^11,15^ although the source of this information is not clear. The frequent use of medical and related mental health services often takes a toll on families, both physically and emotionally, and is also linked to high societal costs.^16^ Therefore, it is imperative to develop a better understanding of the mechanisms that contribute to CPSP and identify potentially modifiable risk factors that will lead to the development of successful prevention and intervention strategies.

### Pediatric Anxiety and Postsurgical Pain

Preoperative anxiety is associated with many adverse clinical, psychological, and behavioral outcomes.^17^ Children with higher levels of preoperative anxiety experience a more complicated perioperative course compared to children with lower levels of preoperative anxiety. This includes a lengthier induction of anesthesia and higher requirements for anesthetic medication.^18–20^ Children with high levels of preoperative anxiety also report higher levels of postoperative pain intensity and require higher doses of pain medications after surgery compared to children with low levels of preoperative anxiety. Compared to children with low levels of state anxiety, children with higher levels of state anxiety have 3.5 times the risk of developing adverse postoperative outcomes, including emergence delirium and maladaptive postoperative behaviors, leading to increased distress during the recovery period.^17,19–21^ Overall, this leads to poorer recovery and longer hospital stays compared to children with low levels of anxiety.

A 2013 systematic review conducted by Chieng et al. examined the relation between perioperative anxiety and postoperative pain in children and adolescents (aged 5–18 years) undergoing elective surgeries (i.e., tonsillectomy, adenoidectomy, and major orthopedic).^6^ Significant moderate positive correlations (*r* = 0.37 to 0.57) between preoperative anxiety and acute postsurgical pain intensity were found; specifically, in one study, children (aged 5–12 years) in the high preoperative anxiety group had higher pain scores and consumed more opioid (i.e., codeine) and nonopioid (i.e., acetaminophen) drugs after discharge than those in the low preoperative anxiety group.^20^ Moreover, significant low-to-moderate positive correlations (*r* = 0.27 to 0.55) between acute postoperative anxiety and postsurgical pain intensity were found up to 4 days after surgery. Overall, this systematic review provided some evidence that children and adolescents who had higher levels of pre- and postoperative anxiety reported higher acute postsurgical pain intensity for up to 14 days after surgery. Since that review, several more recent studies have examined the relations between perioperative anxiety, acute pain, and CPSP.^3,10,22–30^

#### Fear Avoidance Model and Interpersonal Fear Avoidance Model of Pediatric Pain

There is growing evidence implicating other anxiety-related psychological factors in the development of pediatric postsurgical pain. In the pediatric pain population, a fear-avoidance model has been postulated by Asmundson et al.^31^ that asserts that a child can enter a self-perpetuating cycle of chronic pain, avoidance behaviors, and hypervigilance. This is caused by predisposing psychological factors such as fear of pain, anxiety, pain catastrophizing, negative appraisals, negative affectivity, anxiety sensitivity, etc. Parental cognitive, affective, and behavioral factors also fed into child fear avoidance. This cycle further results in more pain intensity, catastrophizing, and functional disability, as demonstrated in a study by Simons and Kaczynski^32^ that used pediatric data to test the adult fear avoidance model. A related model, the interpersonal fear-avoidance model by Goubert and Simons,^33^ builds upon the fear-avoidance model in maintaining pediatric chronic pain. In addition to the self-perpetuating cycle, the interpersonal fear-avoidance model described bidirectional interactions between child and parent factors in that child escape and avoidance behaviors can directly affect parent psychological responses (i.e., pain catastrophizing, pain-related fears) and, in turn, indirectly impact parent pain management behaviors (i.e., overprotectiveness, minimizing).^31–33^ Based on these models, psychosocial constructs such as pain catastrophizing, anxiety sensitivity, pain anxiety, and fear of pain are associated with postsurgical pain.^31,32^ Several studies, which we review in this article, have demonstrated associations between child pain catastrophizing, anxiety sensitivity, and pain anxiety in relation to CPSP.^10,27,34^ Thus, further understanding the role of these anxiety-related factors in pediatric postsurgical pain across the acute and chronic phases might potentially identify a modifiable factor affecting the course of postsurgical pain and help to identify and treat children who are at risk for CPSP.

In addition to child-related factors, parental factors such as parental anxiety, parental catastrophizing, and parental pain anxiety may be associated with pediatric postsurgical pain. Parental anxiety is identified as one of the major modifiable risk factors for children’s preoperative anxiety.^35^ High parental anxiety, both mothers’ and fathers’, is highly correlated with their children’s anxiety up to 6 months after surgery.^36^ Parental anxiety is associated with a host of adverse perioperative outcomes such as an increase in child pain intensity levels, analgesic usage, prolonged anesthetic induction, and recovery time.^21,35^ In general, mothers are found to exhibit a significantly higher level of anxiety than fathers.^37^

In terms of parental catastrophizing, a recent systematic review concluded that parental pain catastrophizing, but not child pain catastrophizing, was predictive of postsurgical pain in children and adolescents undergoing surgery.^1^ However, the findings are only based on two small studies. Therefore, this narrative review will extend the literature by further discussing recent studies on both child and parental anxiety-related psychological factors in predicting postsurgical pain.

## Methods

Because this is a narrative review of published literature, informed consent and research ethics board approval were not obtained.

### Search Strategy

A search of electronic databases CINAHL, PubMed, Scopus, PsycINFO, Mednar, Trip Database, ProQuest Dissertations and Theses, Scirus ETD Search, and Web of Science was conducted from 2013 to the present. The search used the keywords “adolescents,” “anxiety,” “pain,” “child,” “surgery,” and “correlation/relationship.” The exact search terms and search limiters differed between databases due to the availability of search options. Broad search terms were used to ensure that all studies meeting the inclusion criteria were captured in initial searches. In total, 194 studies were collected and screened by title and abstract. Finally, 12 studies were retained for inclusion in this review. Refer to Table 1 for summary of individual study characteristics. Anxiety-related variables are defined in Table 2.

**Table 1.** Individual study characteristics

				Time of assessment		Anxiety-related factors				
Study	Sample size (*n*)	Age range (years)	Surgery type	Acute pain	CPSP	Child anxiety	Child catastrophizing	Parent anxiety	Parent catastrophizing	Summary of results
Birnie et al.^22^	167	10–18	Spinal fusion	6.5 weeks			√		√	Child pain catastrophizing was positively associated with child pain at acute follow-up (*P* < 0.01). Parent pain catastrophizing was not associated with child pain at acute follow-up (*P* > 0.05)
Chidambaran et al.^23^	144	10–18	Spinal fusion	1–2 days	2–3 months 12 months	√	√	√	√	Child preoperative anxiety was positively associated with pain (state; *r* = 0.23, *P* = 0.021) 1–2 days after surgery. Parent preoperative anxiety was not associated with child pain after surgery (state; *P* > 0.05). Child pain catastrophizing was not associated with pain after surgery (*P* > 0.05). Parent pain catastrophizing was not associated with child pain after surgery (*P* > 0.05). The Childhood Anxiety Sensitivity Index (trait; *P* = 0.002) predicted chronic postsurgical pain.
Connelly et al.^24^	50	11–17	Spinal fusion		6 months	√				Children with higher baseline anxiety had a significantly slower improvement in postoperative pain (state; *P* = 0.05).
Esteve et al.^25^	102	9–13	Elective outpatient	Immediately after sugery 24 h after surgery		√	√	√	√	Child anxiety sensitivity was associated with child pain immediately after surgery (trait; *P* < 0.05). Child catastrophizing was associated with child pain immediately after surgery (*P* < 0.05). Caregivers’ anxiety sensitivity was associated with child pain immediately after surgery (trait; *P* < 0.05). Caregivers’ catastrophizing was associated with child pain immediately after surgery (*P* < 0.05).
Pagé et al.^26^	83	8–18	Major^a^	2 weeks	12 months later	√	√	√	√	Parent and child pain anxiety scores 2–3 days after surgery were predictive of pain intensity 2 weeks after surgery. Parent pain catastrophizing scores 2–3 days after surgery predicted child pain intensity reports 1 year later.
Pagé et al.^27^	83	8–18	Major^a^		6 and 12 months	√				Child anxiety sensitivity at 6 months predicted the maintenance of moderate/severe CPSP from 2 months after surgery (trait).
Rabbitts et al.^28^	60	10–18	Major^b^	2 weeks		√	√		√	Parental pain catastrophizing (β = 0.28, *P* < 0.05) was significantly associated with children’s mean pain intensity 2 weeks after surgery.
Rabbitts et al.^10^	60	10–18	Major^b^		12 months	√	√		√	Parental pain catastrophizing before surgery significantly predicted membership in the late recovery group (*P* = 0.03), whereas child catastrophizing and baseline pain did not (*P*s < 0.05).
Rabbitts et al.^38^	119	10–18	Major^b^	2 weeks	4 month	√	√		√	Child anxiety symptoms and pain catastrophizing were not associated with pain after surgery (trait; *P* > 0.05). Parent pain catastrophizing was not associated with child pain after surgery (*P* > 0.05)
Rosenberg et al.^29^	71	≤18 months	Craniofacial surgery	Every 4 h over the first 24 h or until discharge				√		High parental anxiety was correlated with significantly higher pain scores (state; *P* = 0.045)
Rosenbloom et al.^3^	265	8–17	Major^c^		6 and 12 months	√				General anxiety variable (anxiety sensitivity + general anxiety) predicted pain intensity trajectories and pain unpleasantness trajectory group memberships. Parental catastrophizing and anxiety sensitivity were not associated with pain intensity trajectory group membership (trait).
Rullander et al.^30^	37	13–18	Scoliosis surgery	3 days		√				Preoperative anxiety before surgery was not correlated with postoperative pain on day 3. Postoperative pain on day 3 correlates positively with anxiety 6 to 8 months after surgery (state).

^a^Scoliosis, osteotomy, plate insertion tibial/femur, open hip reduction, hip capsulorrhaphy, thoracotomy, thoracoabdominal surgery, Nuss or Ravitch surgical procedure to correct pectus excavatum, sternotomy, laparotomy.

^b^Spine or chest wall surgery.

^c^Osteotomy, plate insertion tibial/femur, surgery for scoliosis, thoracotomy, thoracoabdominal surgery, Nuss/Ravitch pectus repair, sternotomy, laparotomy, laparoscopic-assisted; colectomy, ileostomy, J-pouches.

**Table 2.** Definitions of anxiety-related variables

Variable	Definition	cMeasure(s)
Perioperative anxiety	Excessive worry and nervousness; feelings of tension and apprehension before and after surgery	Modified Yale Preoperative Anxiety Scale^39^ Children’s Perioperative Multidimensional Anxiety Scale^40^
Catastrophizing	The tendency to magnify the threat value of pain and to feel helpless in the context of pain^17^	Pain Catastrophizing Scale for Children^41^ Parent Pain Catastrophizing Scale^42^
Anxiety sensitivity	The degree to which one interprets anxiety-related symptoms as being associated with potentially harmful somatic, psychological, or social consequences^43^	Childhood Anxiety Sensitivity Index^43^ Parent Anxiety Sensitivity Index^44^
State anxiety	A temporary condition experienced in a specific situation^17^	State–Trait Anxiety for Children^45^ State–Trait Anxiety for Youth^46^
Trait anxiety	A general tendency to perceive situations as threatening^17^	State–Trait Anxiety for Children^45^ State–Trait Anxiety for Youth^46^

## Results

### Child Perioperative Anxiety

#### Acute Postsurgical Pain

A study conducted by Rullander et al.^30^ examined the role of stress among adolescents (aged 13–18 years) before and after scoliosis surgery and did not find an association between preoperative anxiety and postsurgical pain 3 days after surgery. However, this study reported a positive correlation between acute postoperative pain and anxiety (using the Trauma Symptom checklist) 6 months after surgery. Chidambaran et al.^23^ reported a positive relation between preoperative anxiety scores and postoperative pain 1 and 2 days after surgery. However, this study utilized a nonvalidated measure of child anxiety, and in the absence of information comparing this measure to a validated measure, it was difficult to interpret their data. Rabbitts et al.^38^ found that anxiety symptoms were not associated with postsurgical pain at 2 weeks in adolescents (aged 10–18 years) undergoing musculoskeletal surgery.

#### Chronic Postsurgical Pain

Chidambaran et al.^23^ found that preoperative anxiety (as measured by a nonvalidated measure) was not associated with postsurgical pain at 1 year in adolescents (aged 10–18 years) undergoing spinal fusion. Rabbitts et al.^38^ also reported a nonsignificant relation between anxiety symptoms and postsurgical pain at 4 months in adolescents (aged 10–18 years) undergoing musculoskeletal surgery.

#### CPSP Pain Trajectories

Connelly et al.^24^ showed that baseline preoperative state anxiety predicted slower postoperative pain resolution and highest reported pain over 6 months in adolescents (aged 11–17 years) after scoliosis surgery.

### Child Anxiety Sensitivity

#### Acute Postsurgical Pain

Esteve et al.^25^ reported that child anxiety sensitivity was associated with higher levels of pain immediately after elective outpatient surgery (*P* < 0.05).

#### Chronic Postsurgical Pain

Chidambaran et al.^23^ showed that adolescent anxiety sensitivity as measured by Childhood Anxiety Sensitivity Index, a validated measure of the belief that anxiety symptoms have negative consequences, at 2 to 3 days predicted CPSP at 12 months after spinal fusion surgery. The odds of pain persistence at 1 year or more was 1.24 times higher for every unit increase in anxiety sensitivity score. Pagé et al.^27^ found that anxiety sensitivity as measured by the Childhood Anxiety Sensitivity Index^43^ at 6 months^44^ was predictive of the maintenance of moderate to severe CPSP 1 year after surgery in youth who underwent orthopedic or general surgeries.

#### CPSP Pain Trajectories

Rosenbloom et al.^3^ used a composite measure of pain-related anxiety and worry based on several validated measures. They found that both baseline pain-related and non-pain-related anxiety and worry were associated with pain intensity trajectories in youths over a period of 12 months.

### Child Pain Catastrophizing

#### Acute Postsurgical Pain

Esteve et al.^25^ and Birnie et al.^22^ both found that an increase in youths’ level of catastrophizing was associated with high pain intensity after surgery. Chidambaran et al.^23^ reported that child pain catastrophizing was not associated with pain at 2 to 3 months in children and adolescents (aged 10–18 years) after spinal fusion surgery.

#### Chronic Postsurgical Pain

Chidambaran et al.^23^ showed that child pain catastrophizing was not associated with postsurgical pain at 1 year in children and adolescents (aged 10–18 years) undergoing spinal fusion.

#### CPSP Pain Trajectories

A study conducted by Rabbitts et al.^10^ showed that pain catastrophizing before surgery was not predictive of pain trajectories in youth.

### Child Pain Anxiety

#### Acute Postsurgical Pain

Pagé et al.^26^ reported that child pain anxiety scores 2 to 3 days after surgery were predictive of youths’ pain intensity 2 weeks after surgery.

#### Chronic Postsurgical Pain

We were unable to identify any studies that examined the relation between child pain anxiety and CPSP.

#### CPSP Pain Trajectories

We were unable to identify any studies that examined the relation between child pain anxiety and CPSP pain trajectory.

### Child Fear of Pain

We were unable to identify any studies that examined the relation between child fear of pain and pediatric postsurgical pain.

### Parental Anxiety

#### Acute Postsurgical Pain

Rosenberg et al.^29^ showed that high parental anxiety as measured by the Hospital Anxiety and Depression Scale^47^ was correlated with significantly higher pain scores in infants and toddlers (mean age 6 months) undergoing cleft lip or palate repair or cranial vault repair. Chidambaran et al.^23^ showed that parental preoperative anxiety was not associated with youths’ pain after surgery (*P* > 0.05).

#### Chronic Postsurgical Pain

We were unable to identify any studies that examined the relation between parental anxiety and CPSP.

#### CPSP Pain Trajectories

Rosenbloom et al.^3^ reported that parental anxiety sensitivity was not associated with youths’ pain intensity trajectories.

### Parent Anxiety Sensitivity

#### Acute Postsurgical Pain

Esteve at al.^25^ showed that caregiver anxiety sensitivity was associated with youths’ pain immediately after surgery (*P* < 0.05).

#### Chronic Postsurgical Pain

We were unable to identify any studies that examined the relation between parental anxiety sensitivity and CPSP.

#### CPSP Pain Trajectories

We were unable to identify any studies that examined the relation between parental anxiety sensitivity and CPSP pain trajectories.

### Parent Pain Catastrophizing

#### Acute Postsurgical Pain

Rabbitts et al.^10^ and Esteve et al.^25^ reported that parent and caregiver catastrophizing before surgery predicted youths’ pain intensity during the acute phase at 24 h and 2 months after surgery. On the other hand, Chidambaran et al.^23^ and Birnie et al.^22^ reported that parent pain catastrophizing was not associated with youths’ acute postsurgical pain at 1 to 2 days and 6.5 weeks after surgery.

#### Chronic Postsurgical Pain

Pagé et al.^26^ reported that parent pain catastrophizing scores 2 to 3 days after surgery predicted youths’ pain intensity reports 1 year later. Chidambaran et al.^23^ found that parent catastrophizing was not associated with postsurgical pain at 1 year in youth (aged 10–18 years) undergoing spinal fusion.

#### CPSP Pain Trajectories

Rabbitts et al.^10^ showed that parental pain catastrophizing before surgery significantly predicted youths’ membership in the late recovery group (*P* = 0.03). Rosenbloom et al.^3^ reported that parental catastrophizing was not associated with pain intensity trajectory group memberships.

### Parent Pain Anxiety

#### Acute Postsurgical Pain

Pagé et al.^26^ reported that parent pain anxiety scores 2 to 3 days after surgery interacted significantly to predict pain intensity 2 weeks after discharge from hospital. Specifically, among parents with high pain anxiety, child pain intensity scores were significantly higher compared to parents with low pain anxiety.

#### Chronic Postsurgical Pain

We were unable to identify any studies that examined the relation between parental pain anxiety and CPSP.

#### CPSP Pain Trajectories

We were unable to identify any studies that examined the relation between parental pain anxiety and CPSP pain trajectory.

### Parent Fear of Pain

We were unable to identify any studies that examined the relation between parent fear of pain and pediatric postsurgical pain.

## Discussion

The aim of this narrative review was to summarize the literature on children’s perioperative anxiety, parental anxiety, and anxiety-related characteristics in relation to pediatric acute postsurgical pain, CPSP, and CPSP trajectories. Overall, the findings seem to suggest that certain child and parental anxiety and anxiety-related factors (i.e., anxiety sensitivity, catastrophizing) are predictive of pediatric postsurgical pain across different phases of the perioperative period. We also found that none of the included studies have examined the relations between fear of pain/pain anxiety and pediatric postsurgical pain.

Compared to a systematic review conducted by Chieng et al.,^6^ our findings suggest that the relation between preoperative anxiety and acute postsurgical pain in children and adolescents undergoing elective surgeries remains unclear. We have extended the literature by including other anxiety-related constructs and found that child anxiety sensitivity appears to be associated with CPSP. There are conflicting results on parental anxiety and parental pain catastrophizing in relation to child acute postsurgical pain, and information is generally lacking beyond the acute phase. This is likely due to the selection of nonvalidated tools and inadequate sample sizes in some studies. More research needs to be done to examine the relation between child and parental anxiety-related factors (i.e., pain anxiety and fear of pain) and postsurgical pain during the chronic phase using validated tools and adequate sample sizes.

### Intervention Studies

Previous research has shown that inadequate preoperative preparation for ambulatory surgeries greatly increases children’s and adolescents’ perioperative anxiety and, as a result, increases the risk of developing postsurgical pain.^6^

Preoperative sedative medications such as midazolam have been routinely used in an attempt to reduce pediatric perioperative anxiety; however, sedatives are associated with many side effects and negative postoperative outcomes (e.g., delirium and pain, delayed emergence from anesthesia).^48,49^ Nonpharmacological interventions have been found to be as effective as pharmacological interventions in reducing pediatric perioperative anxiety.^50^ Specifically, a systematic review conducted by Chow and colleagues suggested that audiovisual (AV) interventions such as preparation videos, multifaceted programs, and interactive games are most effective in reducing youths’ perioperative anxiety.^49^ A mental imagery intervention in the immediate postoperative period was found to be effective in reducing both postoperative anxiety and postsurgical pain levels in children aged 7 to 12 years undergoing ear–nose–throat surgeries.^51^ However, Internet preparation did not reduce pain intensity 2 h after leaving the postanesthesia care unit compared with the standard preparation condition in children and adolescents (aged 10–16 years) undergoing ear–nose–throat surgeries.^52^

A more recent systematic review conducted by Davidson and colleagues also showed that psychological interventions were effective in reducing self-reported acute pain in children and adolescents.^53^ Similar to Chow et al.’s study,^49^ distraction/imagery interventions were shown to be effective in reducing self-reported acute pain, whereas preparation/education interventions were not effective. Thus far, interventions have only focused on the prevention of acute, but not chronic, postsurgical pain. At present, we were unable to identify any studies that examined the impact of perioperative psychological interventions on CPSP and its trajectories.

Another recent systematic review conducted by Chow et al. suggested that AV interventions such as preoperative preparation videos have modest, positive effects on both parental and pediatric preoperative anxiety.^35^ In terms of pediatric postoperative pain, only two studies were pooled to examine the effect of AV interventions directed at parents, and no significant effect of parental AV interventions^54,55^ was found on nurse-rated pain.

Lastly, a study conducted by LaMontagne et al.^56^ assessed the effects of three cognitive–behavioral interventions for reducing postoperative anxiety and pain in adolescents after spinal fusion surgery. These authors found that video intervention with information plus coping instruction was most effective for reducing postoperative anxiety in adolescents with higher preoperative anxiety. Specifically, coping instruction led to a decrease in postoperative anxiety and pain for adolescents younger than 13 years of age.

### Potential Mechanisms

Although this narrative review and other systematic reviews have established a link between anxiety and postsurgical pain, the mechanisms remain unknown. Additional research using the pediatric fear-avoidance model and the interpersonal fear-avoidance model is required to understand relations between anxiety (and its related states such as fear of pain and pain anxiety) and CPSP in order to further understanding of the development of CPSP in children and adolescents. In addition, Pagé et al.^26^ showed how the parent–child measures—which overall were very poorly correlated—tended to increase with time. This has implications for the mechanisms by which children and parents influence each other as time progresses (i.e., they seem to become progressively more in tune with each other). Pagé et al.^26^ also showed an interesting two-way interaction in which child and parent anxiety in the first 2 days after surgery interacted in relation to child functional disability 2 weeks after surgery. This suggests that certain children (low in pain anxiety) may be attempting to protect their parents (high in pain anxiety) and avoid distressing them (and/or avoid being bothered by their parents) by underreporting their pain.

## Conclusion

The purpose of this review was to provide a summary of the current state of knowledge on the role of anxiety in pediatric postsurgical pain. In contrast to the adult literature, the recent pediatric literature did not provide sufficient evidence to support a relation between perioperative anxiety and postsurgical pain in the pediatric population.^6,13^ However, other related psychological factors such as anxiety sensitivity and catastrophizing were found to be predictive of pediatric CPSP and pain trajectories. Research suggests that anxiety sensitivity, not perioperative anxiety, may be predictive of chronic pain after surgery. Parental anxiety also predicted both acute and CPSP in children. These studies revealed the complex relation and interconnectedness between child anxiety, parental anxiety, catastrophizing, and postsurgical pain in children and adolescents undergoing surgery.

Current research seems to support the idea that trait-like factors such as anxiety sensitivity and catastrophizing play an important role in the development and maintenance of CPSP.^27^ It is possible that different manifestations of anxiety (i.e., state vs. trait) across different perioperative phases may play differential roles in the experience of pediatric postsurgical pain. However, the findings on the development and maintenance of CPSP at present are sparse; more research is needed to help further elucidate these relations.

Finally, the literature suggests that AV interventions (e.g., preparation videos with information and coping instructions) may be effective in reducing potential modifiable risk factors such as children’s and parents’ preoperative anxiety. The distraction/imagery interventions were found to be effective in reducing acute pain in children. However, the impact of these interventions on pediatric CPSP are inconclusive because none of the studies have assessed long-term pain as an outcome.

### Future Directions

To reduce the prevalence, severity, and impact of perioperative anxiety and postsurgical pain on youth undergoing surgery, there is a need for much larger, multisite, longitudinal trials (extending beyond 1 year). These future studies should use validated measures of baseline anxiety (both state and trait),^40,57^ parental anxiety, anxiety sensitivity, catastrophizing,^39,58^ pain anxiety, and fear of pain (currently lacking in the literature) in relation to acute and chronic postoperative pain. In addition to pain intensity levels, information on the dosages of analgesic (opioid and nonopioid) medications during surgery, postoperatively in the hospital setting, and at home postdischarge should be collected. Future research should include validated and reliable self-report anxiety measures that are specific for the perioperative setting (e.g., Children’s Perioperative Multidimensional Anxiety Scale),^41^ along with pain intensity levels, at various specified time points (i.e., preoperative clinic visits, on the day of surgery, immediately after surgery, 2–3 months after surgery, and 12 months after surgery) to help better understand the onset and course of the relations between anxiety and pediatric postsurgical pain during different phases. Future research should also examine the potential mechanisms driving these relations as postulated by the fear-avoidance model and by incorporating individual child (e.g., temperament and behaviors via behavioral coding)^42,45^ and parental factors (e.g., coping styles)^46^ in pain trajectories to help identify children and adolescents who are at risk for the development of CPSP. Given that child and parental state anxiety are potentially modifiable risk factors, it is imperative to continue to examine the impacts of interventions in reducing both anxiety and pediatric postsurgical pain and to inform the design on the prevention and intervention strategies in optimizing pediatric perioperative care and pain management.
